# Temporal Changes in the Retinal Pigment Epithelium–Bruch's Membrane Complex Thickness After Autologous Retinal Transplantation in Myopic Eyes

**DOI:** 10.1167/iovs.65.12.25

**Published:** 2024-10-17

**Authors:** Shohei Kitahata, Tatsuya Inoue, Shin Tanaka, Jacob Y. H. Chin, Satoru Shinoda, Maiko Maruyama-Inoue, Kazuaki Kadonosono

**Affiliations:** 1 Department of Ophthalmology and Micro-technology, Yokohama City University, Minami-ku, Yokohama, Japan; 2 National Healthcare Group Eye Institute, Tan Tock Seng Hospital, Singapore; 3 Department of Biostatistics, School of Medicine, Yokohama City University, Kanazawa-ku, Yokohama, Japan

**Keywords:** retinal pigment epithelium, macular hole, autologous retinal transplantation, macular edema

## Abstract

**Purpose:**

To investigate the association between the thickness of the retinal pigment epithelium (RPE)–Bruch's membrane (BM) complex and the development of retinal autograft edema as a postoperative complication following autologous retinal transplantation (ART).

**Methods:**

This retrospective study examined data from 28 eyes of 28 patients (14 males, 14 females; mean age, 61.5 ± 19.8 years) who underwent ART and were followed for 1 year. The RPE–BM complex thickness was measured 2000 µm from the fovea using Image J software. Additionally, the graft blood flow was also evaluated by optical coherence tomography angiography and fluorescein angiography.

**Results:**

Macular hole (MH) diameters ranged from 711.2 ± 251.9 µm to 1299.9 ± 333.0 µm, with MH closure achieved in all patients. RPE–BM complex thickness decreased by 4.17 µm at 6 months and 4.34 µm at 1 year, showing significant differences from preoperative measurements (29.88 ± 4.99 µm; 6 months: 95% confidence interval [CI], 1.62–6.71, *P* = 0.0018; 1 year: 95% CI, 2.03–6.65 µm, *P* = 0.00044). The decrease was significantly greater in the edema-positive group (95% CI, –8.33 to –0.82, *P* = 0.020). Furthermore, the rates of ellipsoid zone (EZ) recovery, alignment of neurosensory layers (ANL), and graft reperfusion were lower in the edema-positive group (EZ, *P* = 0.017; ANL, *P* = 0.0098; reperfusion, *P* = 0.039).

**Conclusions:**

After ART, RPE–BM complex thickness decreases, particularly in cases with postoperative edema, suggesting a potential relationship between RPE function and postoperative outcomes, highlighting the importance of monitoring RPE–BM complex thickness after surgery.

Utilizing contemporary vitreoretinal surgical techniques, typically comprised of pars plana vitrectomy, internal limiting membrane (ILM) peeling using a staining agent and gas tamponade, the success rates for closing macular holes (MHs) after initial surgery surpass 90%.^1^^–^^3^ Nonetheless, MHs, particularly those of larger dimensions or those associated with myopia, often demonstrate resistance to conventional treatments, with reported failure rates ranging between 8% and 44%. Recently, autologous retinal transplantation (ART) has emerged as an effective intervention, achieving MH closure and visual acuity improvements. In our previous study, long-term follow-up of ART grafts indicated that approximately 35% of cases exhibited intragraft reperfusion, and around 50% showed recovery of the ellipsoid zone (EZ). Moreover, we observed that the recovery of the EZ was less frequent in patients who developed postoperative retinal autograft edema. Despite these insights, the detailed mechanisms governing intragraft reperfusion and the onset of edema in retinal grafts remain largely unknown.

However, some types of MHs, usually those that are large or associated with myopia, are often refractory to treatment, with the reported rate of treatment failure varying from 8% to 44%.^1^ Recently, it was reported that ART is effective for MH closure and improvement of the visual acuity in patients for whom hole closure cannot be expected to be obtained by conventional methods.^4^^,^^5^ In our previous report, long-term observation of grafts after ART showed intragraft reperfusion in about 35% of cases and recovery of the EZ in about 50% of cases. We also showed that recovery of the EZ was less likely to occur in cases that developed retinal autograft edema postoperatively.^6^ However, the precise mechanisms of graft reperfusion and development of edema in retinal grafts remain unknown.^7^

The retinal pigment epithelium (RPE) is closely involved in the pathogenesis of edema through its function as a blood–retina barrier, the secretion of cytokines, and its pumping function to generate the hydrostatic pressure required for maintenance of the photoreceptors.^8^^,^^9^ In addition, an irregular structure of the RPE could serve as a sign of photoreceptor disturbance in cases with MHs and might indicate irreversible damage to the photoreceptors.^10^ The RPE–Bruch's membrane (BM) complex thickness has also been attracting much attention, as it has been reported to be associated with retinal diseases and pathologies,^11^^–^^13^ and it is an important parameter for quantifying the RPE. Although retinal autograft edema is an important complication seen after ART surgery, the mechanism of its development remains unclear. Therefore, in this study, we investigated the association between the RPE–BM complex thickness and the development of edema as a complication during the postoperative course after ART.

## Methods

### Study Overview

This study was conducted in accordance with the tenets of the Declaration of Helsinki. We obtained the patients’ data from the Yokohama City University Medical Center. The study was conducted with the approval of the research ethics committee of the Faculty of Medicine of Yokohama City University. Each of the participating patients provided written informed consent for storage of their data in the hospital database and use of the data for research.

### Study Design

This retrospective study was conducted on 28 eyes of 28 patients seen at our clinic at Yokohama City University Medical Center, Yokohama, Japan, from 2017 through 2022. All of the patients enrolled in this study were recruited from the retina outpatient clinic of Yokohama City University Medical Center and fulfilled the following criteria: (1) MH was the only disease cause of the anatomical abnormalities of the macula; (2) patients were followed up for at least 12 months at Yokohama City University Medical Center after the ART surgery; (3) patients had no other systemic disorders; and (4) the MH was more than 400 µm in diameter. During the study period, 36 patients underwent ART, of whom eight had not reached the 1-year mark. Among these eight patients, five were lost to follow-up and three had not yet completed a year of follow up. Each patient underwent spectral-domain optical coherence tomography (SD-OCT) and assessment of best-corrected visual acuity (BCVA) at each follow-up visit. SD-OCT was performed before and after the surgery in all the eyes using the SPECTRALIS HRA-OCT (Heidelberg Engineering, Heidelberg, Germany). BCVA was assessed with full subjective refraction using a Landolt C chart, and the values were converted to the logarithm of the minimal angle of resolution units (logMAR) for the statistical analysis. The thickness of the RPE–BM complex was measured before the ART and at 12 months postoperatively.

### Measurement of the RPE–BM Complex Thickness

**Figure 1.**
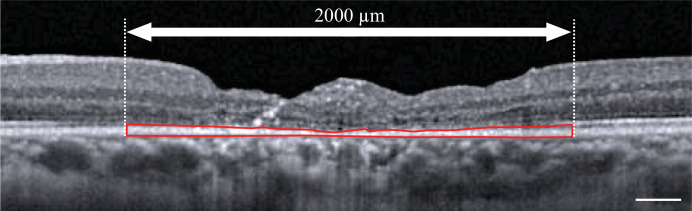
To measure the RPE–BM thickness including the graft and the surrounding retina, we defined an area 2000 µm from the central fovea to the periphery, framed the RPE including the BM in that area, and measured the cross-sectional area using ImageJ. The horizontal and vertical cross-sections were measured and averaged to obtain the average RPE–BM complex area value. The average thickness was defined by dividing the area value by 2000 µm. The scale bar indicates 200 µm.

To measure the RPE–BM complex thickness, including the graft and the surrounding retina, we examined an area 2000 µm from the central fovea toward the periphery, framed the RPE (including the BM in that area), and measured the cross-sectional area using ImageJ software (National Institutes of Health, Bethesda, MD, USA) (Fig. 1).^14^ The horizontal and vertical cross-sections were measured and averaged to obtain the average RPE–BM complex area value. The average thickness was calculated by dividing the area value by 2000 µm.

### Autologous Neurosensory Retinal Transplantation Procedure

The surgery was performed using the method described in a previous report.^5^ All surgeries were performed by the same surgeon, using an identical surgical procedure. After core vitrectomy, a small retinal detachment was created with a microneedle. The entire neurosensory retina was then harvested by bimanual dissection with scissors and forceps. The location of the harvested retinal graft was in the mid-periphery, typically inferior beyond the arcade. The harvested retina was placed over the MH under perfluorocarbon liquid (PFCL) and carefully positioned under the MH with a diamond-dust scraper or FlexLoop (Alcon Laboratories, Fort Worth, TX, USA). The retinal tear created during the harvesting process was subsequently closed by laser photocoagulation. After completion of the transplantation procedure, fluid–gas exchange with sulfur–hexafluoride gas was performed. The patients were positioned face down postoperatively for 3 days.

### Statistical Analysis

Data are expressed as means ± SD, with statistical significance of *P* ≤ 0.05. Statistical analysis was conducted using the SPSS Statistics 22 (IBM, Chicago, IL, USA). A paired *t*-test and one-way ANOVA with Tukey's post hoc pairwise comparisons were performed to compare the results. The comparison of proportions between the two groups was performed using the Fisher's exact test. Each measurement was evaluated by two ophthalmologists. The statistical results are reported with a 95% confidence interval (CI) and *P* value. The intraclass correlation coefficients (ICCs) for the RPE–BM complex thickness are summarized in Table 1.

**Table 1.** ICCs for Each Measurement

Variable	ICC	95% CI
RPE–BM complex thickness before ART (µm)	0.936	0.867 < ICC < 0.970
RPE–BM complex thickness 1-y postoperative (µm)	0.838	0.681 < ICC < 0.921

## Results

**Figure 2.**
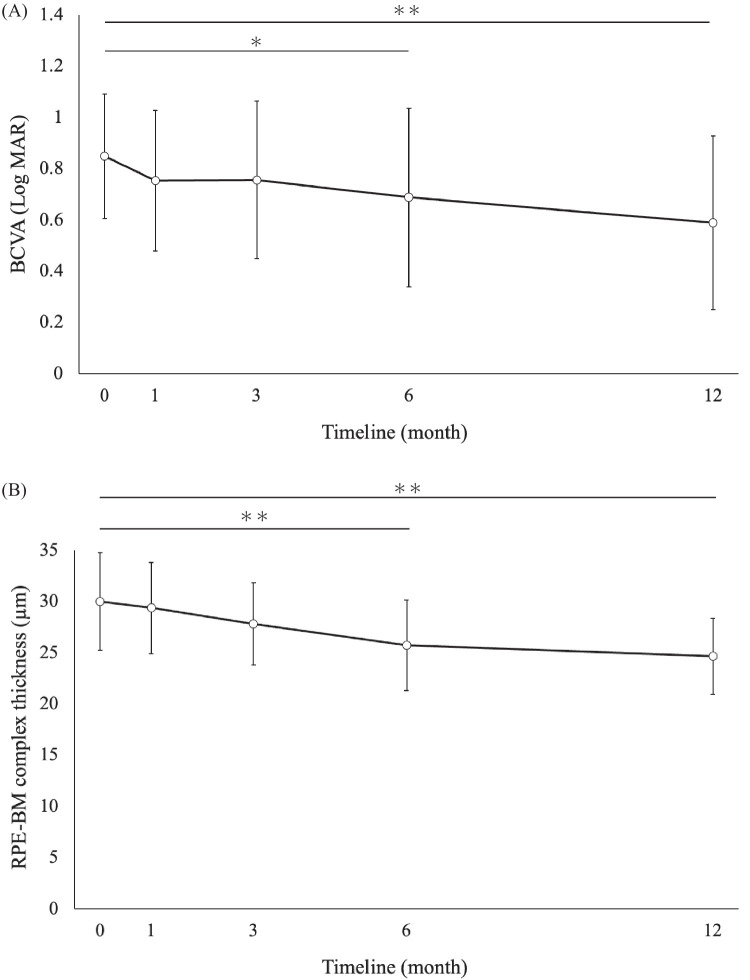
(**A**) The mean BCVA (logMAR) values at 1, 3, 6, and 12 months after autologous retinal transplantation. A one-way repeated-measures ANOVA with Bonferroni correction for post hoc analysis was used for comparison with the values measured at the baseline. *P < 0.05 was considered significant. (**B**) The mean RPE–BM complex thickness values at 1, 3, 6, and 12 months after autologous retinal transplantation. A one-way repeated-measures ANOVA with Bonferroni correction for post hoc analysis was used for comparison with the values measured at the baseline. *P < 0.05 was considered significant.

The characteristics of the 28 patients enrolled in this study are summarized in Table 2. The average follow-up period was 43.3 months. The mean age was 61.5 ± 19.8 years (range, 16–84; 14 men and 14 women). The mean minimum (minimum opening diameter) and maximum (basal diameter) diameters of the MHs were 711.2 ± 251.9 µm and 1299.9 ± 333.0 µm, respectively; the mean BCVA before surgery (baseline) was 0.85 ± 0.24 logMAR (Snellen equivalent, 20/142), and the mean axial length of the eyes was 24.92 ± 3.03 mm, indicating myopia. The minimum MH diameter was 400 to 650 µm in 13 patients and >650 µm in 15 patients. Although good results have been obtained with conventional surgical techniques for MHs measuring up to 650 µm in diameter, in this study the mean minimum diameter of the MH was over 650 µm. Closure of the MH was achieved in all of the treated eyes. The lens status of the eyes remained unchanged after the ART in 13 eyes (46.4%), and intraocular lens insertion was needed in 15 eyes (53.6%). The mean BCVA was 0.75 ± 0.27 (Snellen equivalent, 20/112) at 1 month, 0.76 ± 0.31 (20/115) at 3 months, 0.69 ± 0.35 (20/98) at 6 months, and 0.59 ± 0.34 (20/78) at 1 year after the ART (Fig. 2A). There was a significant improvement at 12 months as compared with the value at the baseline (95% CI, 0.10–0.42, *P* = 0.0018). The mean RPE–BM complex thickness values were 29.88 ± 4.99 µm before the surgery, 29.34 ± 2.45 µm at 1 month, 27.78 ± 4.00 µm at 3 month, 25.71 ± 4.41 µm at 6 month, and 25.54 ± 3.48 µm at 1 year after the surgery (Fig. 2B). The decreases in RPE–BM complex thickness were 4.17 µm at 6 months and 4.34 µm at 1 year. These changes showed a significant difference postoperatively at both 6 months and 1 year compared to preoperative measurements (6 months: 95% CI, 1.62–6.71, *P* = 0.0018; 1 year: 95% CI, 2.03–6.65, *P* = 0.00044). The EZ was restored in 17 eyes (60.7%), and no EZ was evident in 11 eyes (39.3%). At 1 year after the ART, graft reperfusion was seen in 12 eyes (42.9%) and not seen in 18 eyes (57.1%). In addition, by 1 year after the ART, nine patients (32.1%) had developed retinal autograft edema, but 19 patients (67.9%) had no complications. In cases where reperfusion was not achieved, leakage was seen along the surrounding retina. On the other hand, in cases with reperfusion and edema, there was a window defect due to RPE atrophy and hyperfluorescence that increased with time and was observed as leakage. The average onset period of edema was 3.6 months, and the average duration was 11.5 months. Three cases were treated for edema using sub-Tenon's capsule triamcinolone acetonide (STTA) injection. Of these, two cases showed improvement after STTA treatment, but one case had persistent edema.

**Table 2.** Characteristics of Patients Undergoing Autologous Transplantation for Refractory Macular Holes

Characteristic	Value
Patient age (y), mean ± SD	61.5 ± 19.8
Gender, *n* (%)	
Male	14 (50.0)
Female	14 (50.0)
Initial size of macular hole, mean ± SD	
Minimum (µm)	711.2 ± 251.9
Maximum (µm)	1299.9 ± 333.0
Axial length (mm)	24.92 ± 3.03
Visual acuity, preoperative (logMAR)	0.85 ± 0.24
Visual acuity, 1-y postoperative (logMAR)	0.59 ± 0.34
Lens status baseline, *n* (%)	
Phakic, *n* (%)	24 (85.7)
Pseudophakic, *n* (%)	4 (14.3)
RPE–BM complex thickness before ART (µm), mean ± SD	29.88 ± 4.99
RPE–BM complex thickness 1-y postoperative (µm), mean ± SD	25.54 ± 3.48
Lens status in operation, *n* (%)	
Insert	15 (53.6)
Unchanged	13 (46.4)
Postoperative ellipsoid zone in graft, *n* (%)	
Positive	16 (57.1)
Negative	12 (42.9)
Postoperative reperfusion in graft, *n* (%)	
Positive	12 (42.9)
Negative	16 (57.1)
Macular edema in graft, *n* (%)	
Positive	9 (32.1)
Negative	19 (67.9)

**Figure 3.**
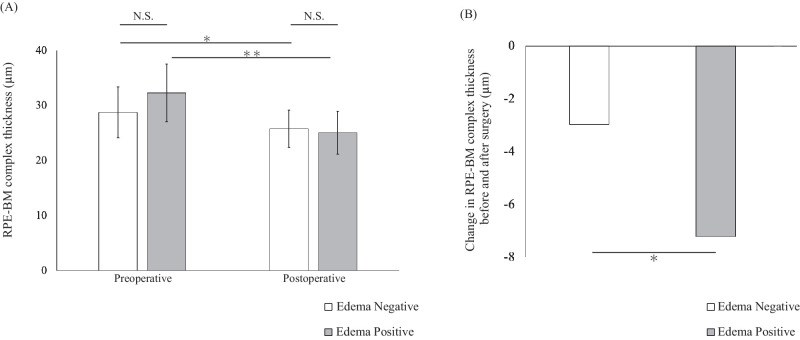
(**A**) RPE–BM complex thickness before and 1 year after surgery with and without retinal autograft edema. A paired *t*-test was used for comparison. *P < 0.05 was considered significant. (**B**) Amount of change in RPE–BM complex thickness before and after surgery; a greater reduction of the RPE–BM complex thickness was observed in the edema-positive group. A paired *t*-test was used for comparison. *P < 0.05 was considered significant.

**Figure 4.**
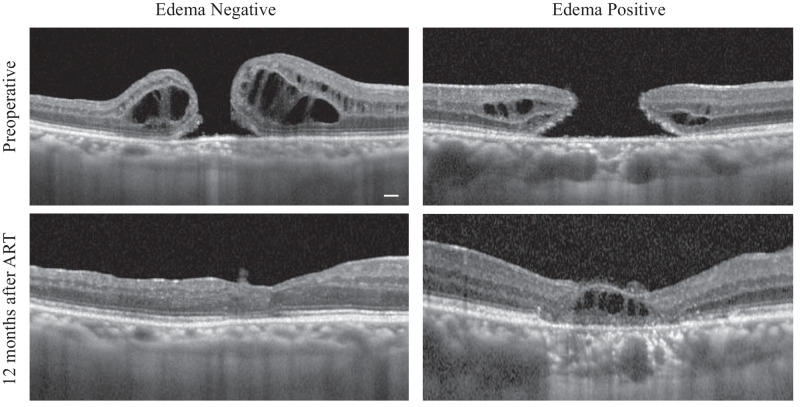
Images on the *left* represent cases without retinal autograft edema., and images on the *right* represent cases that developed retinal autograft edema. In regard to the RPE–BM thickness, it can be seen that the RPE–BM thickness in the images on the *left* (edema-negative group) showed no difference between before and after the surgery, whereas the RPE–BM thickness appears to have decreased after the surgery in the images on the right (edema-positive group). The scale bar indicates 200 µm.

In particular, we focused on development of retinal autograft edema as a complication, and we analyzed the patients by dividing them into two groups according to whether they did or did not develop the complication of retinal autograft edema (edema-positive and edema-negative groups, respectively). We defined the edema-positive group as those who developed edema at least once during the course of observation. There were no significant differences in the mean age of the patients, mean diameter of the MH, mean axial length of the eyes, or pre/postoperative BCVA between the edema-positive and edema-negative groups (Table 3). The mean maximum (basal) diameter of the MH was 1258.9 ± 346.9 µm, and the mean minimum diameter was 754.5 ± 286.1 µm in the edema-negative group, whereas the corresponding values were 1386.4 ± 301.7 µm and 619.8 ± 127.6 µm in the edema-positive group. The axial length of the eye was 25.42 ± 3.41 mm in the edema-negative group, and it was 23.87 ± 1.75 mm in the edema-positive group. The proportion of patients who underwent cataract surgery simultaneously included eight cases (42.1%) in the edema-positive group and seven cases (77.8%) in the edema-negative group, with no significant difference between the groups (*P* = 0.13, Fisher's exact test). The RPE–BM complex thickness values before and after the ART were 28.74 ± 4.59 µm and 25.77 ± 3.37 µm, respectively, in the edema-negative group, and 32.28 ± 5.22 µm and 25.06 ± 3.86 µm, respectively, in the edema-positive group. There was a significant difference in the degree of change of the RPE–BM complex thickness after the surgery as compared with that at the baseline between the two groups, with the decrease in thickness being significantly greater in the edema-positive group (95% CI, –8.33 to –0.82; *P* = 0.020) (Figs. 3A, 3B). Representative cases are shown in Figure 4. The edema-positive group also showed significantly lower rates of graft EZ recovery (*P* = 0.017, Fisher's exact test), alignment of neurosensory layers (ANL; *P* = 0.0098, Fisher's exact test), and graft reperfusion (*P* = 0.039, Fisher's exact test). There was no significant difference in the visual acuity at 1 year postoperatively between patients with and without retinal autograft edema. When the patients were compared by the presence or absence of EZ recovery, there was a significant difference in the 1-year visual acuity after the ART between the EZ-negative group (0.74 ± 0.49) and EZ-positive group (0.30 ± 0.33), whereas there was no difference between the two groups before the surgery. In the 28 eyes included in this study, postoperative cataracts were observed in two cases. No complications such as endophthalmitis, retinal detachment, or non-closure of macular holes were noted. Additionally, in all cases, the PFCL was completely removed, and no residual liquid was observed under the retina.

**Table 3.** Comparison of With and Without Complications of Postoperative Retinal Autograft Edema

	Edema Negative (19 Eyes)	Edema Positive (9 Eyes)	*P*
Patient age (y), mean ± SD	58.1 ± 22.7	68.9 ± 8.7	0.18
Initial size of macular hole (µm), mean ± SD			
Minimum	754.5 ± 286.1	619.8 ± 127.6	0.19
Maximum	1258.9 ± 346.9	1386.4 ± 301.7	0.35
Axial length (mm), mean ± SD	25.42 ± 3.41	23.87 ± 1.75	0.21
Preoperative visual acuity (logMAR), mean ± SD	0.85 ± 0.27	0.84 ± 0.19	0.94
Ratio of idiopathic and post-ILM peeling cases	15:4	6:3	0.49
Cataract surgery, *n* (%)	8 (42.1)	7 (77.8)	0.13
RPE–BM complex thickness (µm) before ART, mean ± SD	28.74 ± 4.59	32.28 ± 5.22	0.10
RPE–BM complex thickness (µm), 1-y postoperative, mean ± SD	25.77 ± 3.37	25.06 ± 3.86	0.64
Change in RPE–BM complex thickness (µm), mean ± SD	−2.97 ± 3.94	−7.22 ± 5.93	0.02
Postoperative visual acuity (logMAR), mean ± SD	0.54 ± 0.38	0.68 ± 0.23	0.46
Postoperative ellipsoid zone in graft, *n* (%)	14 (73.4)	2 (22.2)	0.017
Alignment of neurosensory layers, *n* (%)	10 (52.6)	0	0.0098
Postoperative reperfusion in graft, *n* (%)	11 (57.9)	1 (11.1)	0.039

## Discussion

In this study, a decrease in the mean RPE–BM complex thickness of the graft was observed after ART. The decrease in the RPE–BM complex thickness was significantly greater in the edema-positive group. We also found that the rates of EZ recovery, ANL, and reperfusion of the graft were lower in the edema-positive group.

As we have reported before, in the postoperative period after ART it is important to monitor for the development of retinal autograft edema and ANL in the graft. The fact that we were able to confirm the relationship between the RPE–BM complex thickness and these aforementioned factors is important not only for cases undergoing ART but also for clarifying the pathogenesis of diseases that are closely related to the RPE.

Regarding myopic macular holes, treatments such as ILM inverted flap and amniotic membrane transplantation have also been attempted, and they are generally considered to have a low incidence of complications, such as edema and rejection reactions.^15^^,^^16^ Bamberger et al.^17^ reported postoperative complications associated with amniotic membrane transplantation, including choroidal neovascularization, atrophy, and cystoid macular edema; however, the relationship between complications such as edema and RPE has not been investigated.

The RPE–BM complex thickness has been reported to be increased in cases with dry age-related macular degeneration and decreased by pan retinal photocoagulation for diabetic retinopathy.^13^ The RPE–BM complex thickness has also been reported to be related not only to the above diseases but also to aging, intraocular pressure, race, smoking, and axial length of the eye, and it has attracted attention as a pathogenetic factor.^18^ RPE thinning has been reported to be associated with aging and inflammatory responses. The shape of the RPE nuclei in OXYS rats that were aging were flattened, which is considered to be an aging reaction.^19^ It has also been shown that the mRNA expression of *RPE65* was greatly reduced in aging mice as compared with young mice.^20^ Hence, it might be reasonable to consider that the noted decline in *RPE65* expression with age plays a role in the degeneration of the RPE. These results affirm that aging is associated with overall changes in the choroid–RPE complex.

In fact, the thinning of the RPE layer is thought to be an early sign of development of geographic atrophy (GA). This thinning is considered to be both a precursor to GA formation and an indicator of progression of expanding GA at the edges.^21^^,^^22^ Although there have been no reports on the relationship between RPE–BM complex thickness and MH or ART surgery, there are reports concerning MH and the choroid or RPE. For example, it has been shown in chronic MHs that choroidal thinning occurs and the MH diameter increases.^23^^,^^24^ Additionally, a study observing patients with unoperated MHs over more than 5 years reported atrophy of the RPE.^25^ These findings suggest complex associations among thinning of the RPE, choroid, aging, and MHs. In fact, the postoperative course of grafts has been shown to differ between the inner and outer layers, possibly due to the influence of the retinal and choroidal circulation.^7^ The fact that the inner retina atrophied while the outer retina remained intact suggests that the outer retina is influenced by the choroid and RPE.

Naturally, the RPE has important functions such as degrading spent photoreceptor outer segment fragments and serving as a blood–retina barrier.^26^ The mechanism of development of edema is not yet fully understood, and its causation is thought to be multifactorial, including failure of the pumping system in the RPE, breakdown of the blood–retina barrier, extracellular fluid accumulation, and intraocular inflammation.^8^^,^^27^ In particular, edema is generally thought to be associated with the RPE pumping function, BRB failure, and other RPE-related factors. The greater reduction in RPE–BM complex thickness in the edema-positive group in the present study is consistent with what has been described so far regarding the relationship between RPE and retinal autograft edema.

Although there was no significant difference in the visual acuity between the edema-positive and edema-negative groups, the rates of EZ recovery, ANL, and graft reperfusion were significantly higher in the edema-negative group. The association between retinal autograft edema and postoperative course of a graft cannot be ascribed to any single factor, and multiple factors might be involved. As to why recovery of the EZ is difficult in cases complicated by edema, a similar mechanism is possible, as previous retinitis pigmentosa reports have shown that in the presence of macular edema (ME) developing as a complication, the surrounding photoreceptor cells are reduced and their morphology is disrupted.^28^ Studies examining the effects of diabetic ME on the photoreceptors have suggested that the swelling of retinal cells anterior to the photoreceptors can distort retinal structures and lead to alterations in the regularity and spacing of the cone mosaics.^29^ Our results also showed a lower likelihood of ANL in the presence of retinal autograft edema, and this is thought to be because the layered structure of the graft is disrupted by edema, interfering with its connection to the peripheral retina. Moreover, reperfusion of the graft was also observed more often in the edema-negative than in the edema-positive group. Reperfusion within the graft suggests the development of a vascular anastomosis between the inner layers of the graft and the surrounding retina, and the presence of retinal autograft edema could be expected to make establishment of a vascular anastomosis more difficult.

There was no difference in visual acuity based on the presence or absence of retinal autograft edema; however, considering the impact of edema on photoreceptor cells, a difference may emerge in the long term.^28^ Additionally, the fact that the graft is derived from rod photoreceptors may also contribute to the absence of differences in visual acuity.

One of the limitations of the study was that, although there was a significant difference in the degree of change of the RPE–BM complex thickness between patients who did and did not develop retinal autograft edema as a complication, there was no significant difference in the thickness between before and after the ART (Fig. 3). Although no confounding factors were identified from a clinical perspective, it is possible that unknown confounding factors may have influenced the results of this study. We would like to continue this study with an accumulation of more cases in the future. It still remains unclear if the retinal autograft edema occurred as a result of thinning of the RPE–BM complex or if the RPE–BM complex thickness decreased because of the development of edema. The average duration until the onset of edema was 3.6 months postoperatively, whereas a significant reduction in RPE–BM complex thickness was observed at the 6-month postoperative mark. The relationship between edema and RPE–BM complex thickness is not clear in the early stages. There may be a possibility that edema can be more easily observed and the reduction in RPE–BM complex thickness occurs gradually even in cases of decreased RPE function. We believe that analysis over a longer term is needed.

In conclusion, our study revealed that the RPE thickness decreased after ART, particularly in cases that developed retinal autograft edema as a complication. Our findings suggested that the changes in the RPE–BM complex thickness may be related to the postoperative course after ART. The present analysis was based on changes in the RPE–BM complex thickness after ART, perhaps indirectly suggesting the importance of the RPE function. Monitoring of the RPE–BM complex thickness may become an important tool for following the course of the patients.
